# Supplementary material for: Content-rich biological network constructed by mining PubMed abstracts
Source: BMC Bioinformatics. 2004 Oct 8;5:147. doi: 10.1186/1471-2105-5-147 (PMC528731; doi:10.1186/1471-2105-5-147)
Supplement: Additional File 2 — The original results of the above study (non-essential files are deleted to keep the file size under the limit set by BMC bioinformatics). [file 1471-2105-5-147-S2.bz2 › chilibotAdditionalFile2/dip05/560ID9537587E10921/html/HGFAC_MET.html]

 


 **HGFAC** and **MET** 
  
Found 18 abstracts in PubMed, retrieved 05.  
 

 What does Google say? 
 PDF only 
| .edu only 

---

**Interactive relationship** (e.g. stimulation, inhibition, etc)

**Inhibitory relationship**- Tissue microarray analysis of hepatocyte growth factor  **Met**  pathway components reveals a role for  **Met** , matriptase, and hepatocyte growth factor activator  [ **HGFAC** ]  inhibitor 1 in the progression of node negative breast cancer.  Ref: 12615728 Cancer Res, 2003
- In tumorigenesis and tissue regeneration, HAI 1 regulates the hepatocyte growth factor HGF c  **Met**   [ **MET** ]  pathway by inhibiting the activity of HGF activator  [ **HGFAC** ]  HGFA  [ **HGFAC** ]  and matriptase, two serine proteases that convert pro HGF into its biologically active form.  Ref: 12815039 J Biol Chem< MedlineTA>J Biol Chem, 2003
**Neutral relationship**

**Non-interactive relationship** (e.g. studied together, co-existance, homology, etc.)

- Western blot analyses using antibodies against hepatocyte growth factor, hepatocyte growth factor receptor  [ **MET** ] , phosphotyrosine, hepatocyte growth factor activator  [ **HGFAC** ] , urokinase type plasminogen activator receptor, urokinase type plasminogen activator, plasminogen activator inhibitor 1, fibrinogen and plasmin were performed on membrane enriched fractions from the frozen tissue, as was collagen zymography for matrix metalloproteinase 2 and matrix metalloproteinase 9.  Ref: 12527708 Mod Pathol, 2003
- Although the presence of hepatocyte growth factor and its c  **MET**   [ **MET** ]  receptor have been reported in male mammalian reproductive tracts, our novel findings on the secretion of HGFA  [ **HGFAC** ]  precursor from turkeys may help to elucidate the regulation of activated hepatocyte growth factor.  Ref: 12128063 Comp Biochem Physiol B Biochem Mol Biol, 2002
- To determine whether these factors contribute to the distinct etiologies of fibrolamellar hepatocellular carcinoma and traditional hepatocellular carcinoma, we assayed hepatocyte growth factor, the hepatocyte growth factor receptor  [ **MET** ] , and two hepatocyte growth factor activators, hepatocyte growth factor activator  [ **HGFAC** ]  and urokinase type plasminogen activator, in hepatocellular carcinoma, fibrolamellar hepatocellular carcinoma, cirrhotic liver and normal liver.  Ref: 12527708 Mod Pathol, 2003
- The gene expression of HGF and keratinocyte growth factor KGF in gingival connective tissue, and c  **met**   [ **MET** ] , HGFA  [ **HGFAC** ]  and KGF receptor KGFR in gingival epithelial tissues was monitored using RT PCR.  Ref: 12613501 J Oral Sci, 2002
- To examine the role of the HGF system in epithelial invasion followed by loss of connective tissue attachment in periodontitis, mRNA expression of HGF, its receptor c  **met**   [ **MET** ]  and HGFA  [ **HGFAC** ]  in gingival tissues was monitored.  Ref: 12613501 J Oral Sci, 2002
